# Examining Skills and Abilities During the Pandemic – Psychology Students’ and Examiners’ Perceptions of a Digital OSCE

**DOI:** 10.1177/14757257221114038

**Published:** 2022-07-22

**Authors:** Camilla Hakelind, Anna E. Sundström

**Affiliations:** Department of Psychology, 8075 Umeå University, Sweden; Department of Psychology, 8075 Umeå University, Sweden

**Keywords:** Clinical psychology training, competency assessment, online assessment, simulation-based methods

## Abstract

Finding valid and reliable ways to assess complex clinical skills within psychology is a challenge. Recently, there have been some examples of applying Objective Structured Clinical Examinations (OSCEs) in psychology for making such assessments. The aim of this study was to examine students’ and examiners’ perceptions of a digital OSCE in psychology regarding quality and students’ feelings about the OSCE. Participants were 51 students enrolled in the Programme for Master of Science in Clinical Psychology during two semesters and nine examiners assessing each OSCE occasion, at Umeå University, Sweden. Web-based questionnaires were used for data collection. Psychometric analyses indicated that the subscales in the student questionnaire had adequate or close to adequate levels of item and scale reliability. Both students and examiners felt that the digital OSCE was realistic, valid and well-aligned with professional practice. Although students perceived the digital OSCE as stressful, the results showed that they were focused and concentrated and found the OSCE to be a positive learning experience, implying that the stress did not affect performance to any significant extent. Based on the examiners’ experiences, it can be concluded that there are both advantages and disadvantages which need to be considered when planning future digital OSCEs.

## Introduction

In the field of clinical psychology education there is general agreement about the importance of developing and evaluating clinical core competencies (Kaslow et al., 2009; Pachana et al., 2011; Stevens et al., 2017), such as communication, diagnosis, and therapeutic techniques (Sheen et al., 2015). Identifying valid and reliable ways to assess complex clinical skills within psychology is a challenge. Traditional assessment forms such as case-reports, essays and exams with multiple-choice questions mainly assess knowledge and understanding and provide little information of students’ practical skills and abilities. For example, although students have knowledge about psychotherapeutic techniques such as CBT treatment skills, this does not guarantee that they can apply these techniques in a proper way with clients (Yap et al., 2021). In traditional assessment of clinical competence, educational programs in clinical psychology have traditionally relied on supervisor reports from practice placements (Dunsmuir et al., 2017; Hitzeman et al., 2020). There are, however, several limitations to supervisor assessments, such as the non-standardized format, the dual role of mentor/examiner (which can lead to halo effects), and the difficulty of observing a range of clinical skills and abilities (Dunsmuir et al., 2017; Gonsalvez et al., 2013; Stevens et al., 2017). Despite these limitations, supervisor reports remain the main assessment method of professional competence in clinical psychology training (Dunsmuir et al., 2017). There is therefore a need for more standardized, objective methods to assess professional competence in clinical psychology in a reliable and valid manner.

### Using OSCE to Assess Clinical Competence in Psychology

Within the medical field, the Objective Structured Clinical Examination (OSCE), introduced by Harden et al. (1975) is routinely used to assess clinical competence and is viewed as the gold standard for the assessment (Khan et al., 2013). The OSCE is used to assess students’ clinical competence in a range of simulated conditions where students are given the opportunity to show their competencies, combining live clinical interactions with the standardization of tasks and assessment criteria with multiple observations of each student. The OSCE format involves numerous time-limited practical tasks, each of which usually performed in a room called a “station”, typically with trained actors playing the role of a patient presenting with one or more clinical symptoms. In each station, the student's performance is observed and assessed by an examiner using a checklist and most often also a global rating scale (Khan et al., 2013). The OSCE is recommended as a highly authentic, reliable and valid way of assessing competency (Kaslow et al., 2009), but studies indicate that it is perceived as stressful by students (Brand & Schoonheim-Klein, 2009). Although the OSCE is widely used both in medical training and in other educational contexts such as nursing, dentistry, pharmacy and related areas such as psychiatry (Hodges et al., 2002; Murcott & Clarke, 2017), it has not received as much attention in clinical psychology training programs (Yap et al., 2021). However, a recent study describing the implementation of an OSCE in clinical psychology highlights the benefits of the assessment method in terms of student learning and as preparation for clinical practice (Glatz et al., 2022).

There are a few qualitative and quantitative studies reporting on the use of the OSCE in clinical psychology, and the focus in these studies has mainly been on students’, and to some extent examiners’, perceptions of the OSCE. The content and format of these psychology OSCE examinations differ to some extent. For example, the OSCE described in Yap et al. (2012) consisted of seven stations assessing clinical conditions taught in the preparatory course, including, e.g., major depressive disorder, schizophrenia and borderline personality disorder. The OSCE described in Sheen et al. (2015), on the other hand, comprised four stations assessing skills such as differential diagnosis, history taking and mental state examination. In another study, an assessment similar to the OSCE, called the OSPA (Objective Structured Professional Assessments) was used for assessing educational psychologist students’ professional skills. The OSPA consisted of 4 stations assessing competencies required for practice with young people (Dunsmuir et al., 2017).

Results from the studies evaluating students’ perceptions of these OSCE examinations are rather consistent despite these differences. In general, findings suggest that the OSCE is perceived as a valid, high quality, fair and authentic form of assessment (Dunsmuir et al., 2017; Sheen et al., 2015; Yap et al., 2012). In line with student evaluations of OSCEs within the medical field, the psychology OSCEs are also perceived as stressful by the students (Dunsmuir et al., 2017; Roberts et al., 2017; Sheen et al., 2015; Yap et al., 2012). Several studies report that a great majority of students perceive the OSCE as anxiety provoking (Johnson et al., 2018, Roberts et al., 2017; Yap et al., 2012). These studies have typically focused on how stressful the test situation is perceived and have not included questions regarding, for example, students’ ability to concentrate and focus. Therefore, the present study aimed to include student perceptions of the OSCE test situation with respects to such aspects.

Student and examiner feedback has also been used to guide the implementation of changes in the OSCE procedure (Johnson et al., 2018). Changes made were, for example, related to information about the OSCE and instructions to students as well as formulations regarding feedback from the OSCE. A follow-up indicated that these changes led to a more positive perception of the OSCE format and less test anxiety among students. Despite the high levels of stress related to the OSCE, students also report that the OSCE is a valuable learning experience and that feedback from the OSCE enhances their learning (Roberts et al., 2017; [Bibr bibr24-14757257221114038][Bibr bibr26-14757257221114038]).

Some studies concerning the use of the OSCE in clinical psychology have also included the examiner perspective. Findings from these studies indicate that examiners are overwhelmingly positive towards the OSCE, recommending the inclusion of OSCEs in clinical psychology postgraduate programs (Dunsmuir et al., 2017; Johnson et al., 2018; Sheen et al., 2015). The primary disadvantage noted by the examiners is related to the time and cost associated with the OSCE (Sheen et al., 2015).

### Digital OSCEs During the Covid-19 Pandemic

As a result of the Covid-19 pandemic and its associated lockdowns, universities worldwide were forced to deliver remote teaching and quickly find digital alternatives for assessment. This has affected the arrangements of OSCEs as well, with virtual OSCEs being introduced in medical education (Blythe et al., 2021) and dental education (Hytönen et al., 2021). In Sweden, the OSCE has been implemented in the five year long Programme for Master of Science in Clinical Psychology at Umeå University since 2018. In 2021, due to the pandemic, the OSCE was modified to a digital format. When implementing OSCEs, there are factors inherent in the administration that can affect the validity of the OSCE scores and hence the usability of the assessment (Crooks et al., 1996). For example, low motivation, test anxiety, poor communication of test requirements and inappropriate assessment conditions may have a negative impact on the validity of the OSCE. Although previous studies have examined student and examiner perceptions of OSCE in clinical psychology, there are to our knowledge no previous studies that have examined the perceptions of a digital OSCE in clinical psychology. Furthermore, previous studies have indicated that the OSCE is perceived as stressful by the students. However, little is known about other aspects such as students’ ability to concentrate and focus, and to what extent their feelings of stress and anxiety before and during the OSCE have impacted on their performance and consequently affected the validity of the assessment. In the evaluation of the digital OSCE, students’ and examiners’ perceptions can thus provide important information.

### Aim

The aim of this study was twofold: first, to broadly examine students’ feelings about the digital OSCE and their perceptions of the learning experience and the quality and validity of the digital OSCE. Second, to examine examiners’ experience and perceptions of the quality and validity of the digital OSCE.

## Method

### Procedure

A summative digital psychology OSCE was administered to students in the 6th semester of the five-year Programme for Master of Science in Clinical Psychology, at Umeå University, Sweden in 2021. Within the program, there is only one OSCE, and this examination summarizes and integrates clinical skills taught from semester three to semester six. For the digital OSCE, two parallel circuits of five stations were delivered in Zoom during one day. The stations were designed to represent authentic situations for a professional clinical psychologist and assessed clinical core competencies such as therapeutic skills, diagnostic assessment, testing, and giving psycho-educative information (see Table 1). The content of the stations was chosen to match the curricular goals of the program. In all stations, students encountered standardized patients (SPs). The SPs were given written instructions before the OSCE concerning how to behave as patients (for an example, see Appendix A) and were also personally instructed by the examiner responsible for the station.

**Table 1.** Description of the OSCE stations.

Stations Spring 2021	Student examination task	Patient
CBT technique	Performing a functional analysis	Patient with depressive problems
PDT technique	Build alliance and display empathy	Stressed and tired patient
Psychological testing	Explaining the assessment procedure	Patient with incipient dementia
Diagnostic interview	Performing an open diagnostic interview	Young patient with depressive and anxiety problems
Anamnestic interview	Interviewing parent about the child's problems	Parent of a child with anxiety problems
Stations Autumn 2021		
CBT technique	Situational analysis and explaining Clark's model of anxiety	Patient with anxiety problems
PDT technique	Build alliance and display empathy	Stressed and tired patient
Psychological testing	Administrating cognitive test	Patient with mild concussion
Diagnostic interview	Performing an open diagnostic interview	Young patient with depressive symtoms
MI technique	Performing basic MI techniques	Upset and worried parent seeking support

Note. CBT  =  Cognitive Behavior Therapy, PDT  =  Psychodynamic Therapy, MI  =  Motivational Interviewing.

In each station one examiner observed the student's interaction with the SP for 13 min and assessed the student performance by completing a checklist and a global rating scale (for an example, see Appendix A). The checklists were constructed to include both task-related and general criteria relevant to the psychologist profession, such as the ability to display and express empathy, to keep the structure of conversations, and to collaborate with the SPs. The maximum checklist score at each station was 10 points, and the global rating scale had the following categories; *excellent, clear pass, borderline pass* and *clear fail*. The cut-score of the OSCE was set to 60 percent, which is a faculty-wide standard that is used on most examinations at the department. In addition, a maximum of one clear fail was allowed in order to pass. Students had four minutes of preparation time between each station, during which they read short instructions for the coming station (for an example, see Appendix A). Each station had its own room in Zoom, where the student, the SP and examiner met. Before the OSCE, students were handed documents with the information they needed, such as zoom-links, instructions and exact timepoints for each station. The examiners were provided with corresponding documents. Concerning regulations for re-examination, the same rules apply for the OSCE as for other examinations within the program. Students who fail are entitled to four additional attempts within two years.

Shortly after conducting the OSCE, students and examiners were contacted and asked to answer web-based questionnaires comprising questions about their perception of the OSCE. Two reminders were sent out to students and examiners in the following two weeks after the initial invitation.

### Participants

Out of 75 students completing the digital OSCE in the spring and autumn semester of 2021, 51 students in the ages between 21 and 36, agreed to participate (mean age: 26 (SD  =  3.77); 80% females) in the study. The students reported that they prepared for the OSCE on average 35 h per week (SD  =  13.9). For the first digital OSCE, 9 of 11 examiners agreed to participate, and for the second digital OSCE 9 of 10 examiners agreed to participate in the study. The examiner ratings from the two OSCE occasions were merged to one group, consisting of 18 examiner ratings, for the analyses. Some of the examiners participated on both OSCE occasions. However, since participation in the study was anonymous, we did not have information about which of the examiners that participated on both occasions.

All participants gave their informed consent to participate in the study. Ethical approval was obtained from the Swedish Ethical Review Authority (Dnr 2020–01440).

### Instruments

Webb-based questionnaires were used for data collection and were administered to students and examiners shortly after the OSCE. The questionnaires were based on one originally developed and used by Yap et al. (2012) and later modified by Sheen et al. (2015) and permission to use the questionnaire was obtained. The original student questionnaire comprised questions assessing students’ perceptions of the quality in terms of validity, relevance, realism, and fairness of the OSCE, as well as their experiences of anxiety and views on how well it fit into clinical psychology training. In the present study, questions about students’ feelings before and during the OSCE were also added to the questionnaire in order to obtain more elaborate information about these aspects. The questionnaire used in the present study comprised four subscales. The first subscale included 14 items about students’ feelings and behaviors before and during the OSCE. The second subscale included four items about students’ learning experience in relation to the OSCE. The third subscale consisted of nine items assessing students’ perceptions of the quality of the OSCE, and the fourth subscale comprised nine items about students’ perceptions of the validity of the OSCE. Participants responded to the items on a 5-point Likert scale, ranging from 1 = Strongly disagree to 5  =  Strongly agree. In the student questionnaire the questions were made mandatory, which meant that the respondents had to answer a question before they could move on to the next one. The examiner questionnaire comprised questions about the perceived quality and validity of the OSCE (corresponding to subscale three and four). Because the examiners did not have full insight into all stations, they were provided the option “unable to assess”. In addition to the rating scale items, the questionnaire to the examiners also included three open-ended questions:

– How did you feel that the assessments during the digital OSCE worked for you? Please describe any problems that you experienced or aspects that you think need to be improved to make assessments as accurate as possible.
– Can you see any benefits of using a digital OSCE instead of an onsite OSCE? If so, please describe.
– Can you see any disadvantages of using a digital OSCE instead of an onsite OSCE? If so, please describe.

### Analyses

Descriptive statistics were used to analyze the results from the student and examiner questionnaires. As the psychometric properties of the questionnaire by Yap et al. (2012) and Sheen et al. (2015) have not previously been reported on, the items in the modified student questionnaire used in the present study were subjected to item analysis. The corrected item-total correlation (ITC) was used as discrimination index, and values >.20 were considered satisfactory (Kline, 2015). Cronbach's alpha was used as a measure of internal consistency, and values >.70 were considered acceptable (AERA, APA, NCME, 2014). Because the sample of examiners was small, and there were partially missing values due to the “unable to assess alternative”, only the student version of the questionnaire was subjected to psychometric analysis.

Finally, an inductive content analysis was conducted to analyze examiners’ responses to the open-ended questions about their experience of the digital OSCE. This was done through the process of reading the material thoroughly, coding the material, subsequently creating meaningful content-based categories, while keeping close to the analyzed material. Finally, the material was summarized into categories and subcategories and presented (Elo & Kyngäs, 2008). In the presentation of the results, great care has been taken to maintain the semantic content of the material, i.e., all aspects that the examiners raised have been included.

## Results

In this study, a digital OSCE was examined from a student and an examiner perspective. First, the results concerning the psychometric properties of the student questionnaire are presented, followed by the results regarding students’ perspectives and examiners’ perspectives.

### Student Perceptions

As a first step, the items in the student questionnaire were subjected to item analysis. For the sub-scale about students’ feelings before and during the OSCE, ten out of 14 items had acceptable levels of item discrimination (see Table 2). In the sub-scale about students’ perceptions of the quality of the OSCE, one item had a low item-total correlation. The remaining items had acceptable or close to acceptable levels of item discrimination (see Table 4). The items in the sub-scale about students’ perception of their learning in relation to OSCE and the subscale about validity of the OSCE showed acceptable levels of item discrimination (see Tables 3 and 5). The internal consistency was acceptable or close to acceptable for the subscales in the student questionnaire (se Tables 2–5).

**Table 2.** Descriptive statistics for students' feelings and behavior before and during the OSCE in percent (n = 51), item total correlation (ITC) and Cronbach's alpha.

	Strongly agree	Agree	Agree to some extent	Disagree	Strongly disagree	ITC
I prepared more for the OSCE than I usually do for exams.	31.4	35.3	21.6	9.8	2.0	.20
I felt that the OSCE is a more important examination than other examinations.	60.8	27.5	9.8	2.0	0	.32
I felt excited before the OSCE*	17.6	41.2	35.3	5.9	0	.16
I felt sure that I would pass the OSCE*	5.9	33.3	45.1	9.8	5.9	.01
I was afraid to fail the OSCE	37.3	29.4	19.6	7.8	5.9	.44
I was worried about how difficult the OSCE would be	29.4	41.2	17.6	9.8	2.0	.51
I worried about whether I would have time to perform the task within each station	27.5	19.6	19.6	23.5	9.8	.48
During the OSCE, I felt concentrated*	45.1	37.3	9.8	7.8	0	.22
During the OSCE, I felt calm*	3.9	15.7	29.4	37.3	13.7	.48
During the OSCE, I felt highly focused*	17.6	43.1	25.5	9.8	3.9	-.06
During the OSCE, I felt nervous	39.2	37.3	17.6	5.9	0	.56
During the OSCE, I felt stressed	17.6	41.2	23.5	11.8	5.9	.58
During the OSCE, I felt mentally blocked	5.9	5.9	31.4	39.2	17.6	.49
						Alpha .73

Note: *Reversed scoring for reliability analysis. Average inter-item correlation r  =  .16.

**Table 3.** Descriptive statistics for students' perception of the learning experience in percent (n = 51), item total correlation (ITC) and Cronbach's alpha.

	Strongly agree	Agree	Agree to some extent	Disagree	Strongly disagree	ITC
On the whole, the OSCE is a valuable experience.	86.3	9.8	2.0	2.0	0	.36
Preparing for and conducting the OSCE benefited my learning.	88.2	7.8	2.0	2.0	0	.59
The feedback I received after completing the OSCE benefited my learning.	29.4	39.2	29.4	2.0	0	.52
The feedback I received after completing the OSCE increased self-awareness of my professional skills.	27.5	47.1	17.6	5.9	2.0	.59
						Alpha .72

Note: Average inter-item correlation: r  =  .40.

**Table 4.** Descriptive statistics for students' perceptions of the quality of the OSCE in percent (n = 51), item total correlation (ITC) and Cronbach's alpha.

	Strongly agree	Agree	Agree to some extent	Disagree	Strongly disagree	ITC
Skills in leading therapeutic sessions	74.5	19.6	5.9	0	0	.62
Ability to act professionally	76.5	17.6	5.9	0	0	.45
Ability to respond empathetically	64.7	23.5	11.8	0	0	.43
Ability to convey psychoeducation	60.8	23.5	15.7	0	0	.56
Ability to assess mental health problems	43.1	41.2	13.7	2.0	0	.67
Skills in applying CBT techniques	52.9	37.3	7.8	2.0	0	.67
Skills in applying PDT techniques	45.1	37.3	15.7	2.0	0	.66
Skills in psychological testing	41.2	33.3	13.7	3.9	7.8	.53
Ability to convey results from psychological assessments	39.2	29.4	9.8	5.9	15.7	.48
						Alpha .86

Note: Average inter-item correlation: r  =  .40.

**Table 5.** Descriptive statistics for students' perceptions of validity of the OSCE in percent (n = 51), item total correlation (ITC) and Cronbach's alpha.

	Strongly agree	Agree	Agree to some extent	Disagree	Strongly disagree	ITC
The tasks of each OSCE station were clearly described	43.1	37.3	15.7	3.9	0	.11
The OSCE stations reflected material taken up in teaching in integrative course or in previous courses on the program	49.0	47.1	3.9	0	0	.20
OSCE is a better method for assessing clinical competence compared with written examinations	78.4	15.7	5.9	0	0	.49
The content of the OSCE stations was relevant to the course	64.7	33.3	2.0	0	0	.48
The figurants in the OSCE were realistic	60.8	35.3	3.9	0	0	.22
The stations in the OSCE were relevant in relation to the psychologist's clinical role	58.8	37.3	3.9	0	0	.34
The OSCE is a quality-enhancing element in psychologist education	74.5	19.6	3.9	2.0	0	.54
The OSCE seems to be a fair examination form	47.1	43.1	7.8	2.0	0	.49
After taking the integrative course I felt well prepared for the OSCE	27.5	56.9	15.7	0	0	.39
						Alpha .69

Note: Average inter-item correlation: r  =  .20.

#### Students’ Feelings Before and During the OSCE

Overall, most students felt that the OSCE was a more important examination than other examinations, and the result showed that the students worried before the examination, but also that they felt excited. Over 65 percent were afraid to fail, but on the other hand, nearly 40 percent answered that they felt sure that they would pass the OSCE. About half of the students worried about the time limit. Almost 90% of the students considered the OSCE to be a more important examination than other examinations and over 65% prepared more than they usually do for exams (see Table 2). Regarding students’ feelings during the OSCE, the majority reported that they felt stressed and nervous, but fewer, just over ten percent, felt mentally blocked during the test situation. A majority reported that they felt concentrated and highly focused. However, fewer than 20 percent of the students felt calm during the OSCE (see Table 2).

#### Students’ Perception of the Learning Experience

Concerning students’ perception of the learning experience of taking the integrative course and the OSCE, the students’ responses were very positive. Most of the students thought that several aspects such as preparing for, conducting and getting feedback from the OSCE benefited their learning and that OSCE was a positive learning experience. Moreover, the majority also believed that the feedback from the OSCE increased the self-awareness of their professional skills (see Table 3).

#### Students’ Perceptions of Quality and Validity of the OSCE

Overall, most of the students gave high ratings concerning the quality of the OSCE. Most considered the OSCE to be a fair, quality enhancing and relevant assessment form, and considered the stations relevant and SPs realistic (see Table 5).

The students were also asked about their perceptions of the validity of the OSCE for assessing different clinical skills and abilities (see Table 4). In general, students took a positive view of the validity of the OSCE in relation to assessing the different psychologist core competencies in a valid manner.

### Examiners Perceptions

#### Examiners Perception of Quality and Validity of the OSCE

The examiners’ perception of the fairness, relevance and quality of the OSCE positive were overall positive, and all the participating examiners agreed that the OSCE is a quality-enhancing element in the psychologist education (see Table 6).

**Table 6.** Descriptive statistics for examiners' perceptions of quality of the OSCE in percent (n = 18).

	Strongly agree	Agree	Agree to some extent	Disagree	Strongly disagree	Unable to assess/missing
The tasks of each OSCE station were clearly described	16.7	50.0	5.6	0	0	27.8
The OSCE stations reflected material taken up in teaching in integrative course or in previous courses on the program	55.6	27.8	0	0	0	16.7
OSCE is a better method for assessing clinical competence compared with written examinations	66.7	22.2	11.1	0	0	0
The content of the OSCE stations was relevant to the course	61.1	22.2	16.7	0	0	0
The figurants in the OSCE were realistic	44.4	50.0	0	0	0	5.6
The stations in the OSCE were relevant in relation to the psychologist's clinical role	66.7	27.8	0	0	0	5.6
The OSCE is a quality-enhancing element in psychologist education	88.9	11.1	0	0	0	0
The OSCE seems to be a fair examination form	11.1	66.7	11.1	5.6	0	5.6

The examiners rated the validity of OSCE highest for the general psychologist related skills and abilities such as ability to assess mental health problems, ability to convey psychoeducation, ability to respond empathetically and act professionally and skills in leading therapeutic sessions. Concerning more specific skills, such as skills in psychological testing, and conveying results from such testing, as well as for CBT and PDT techniques, the ratings of validity were considerably lower, and the examiners instead used the alternative “unable to assess” to a much higher degree (see Table 7).

**Table 7.** Descriptive statistics for examiners' perceptions of validity of the OSCE in percent (n = 18).

	Strongly agree	Agree	Agree to some extent	Disagree	Strongly disagree	Unable to assess/missing
Skills in leading therapeutic sessions	55.6	33.3	11.1	0	0	0
Ability to act professionally	38.9	44.4	16.7	0	0	0
Ability to respond empathetically	33.3	44.4	22.2	0	0	0
Ability to convey psychoeducation	50.0	44.4	0	0	0	5.6
Ability to assess mental health problems	22.2	55.6	16.7	0	0	5.6
Skills in applying CBT techniques	27.8	22.2	11.1	0	0	38.9
Skills in applying PDT techniques	5.6	38.9	16.7	5.6	0	33.3
Skills in psychological testing	11.1	16.7	22.2	11.1	0	38.9
Ability to convey results from psychological assessments	11.1	38.9	5.6	0	0	44.4

#### Examiners’ Experiences of the Digital OSCE

Examiners were also asked to comment on their experiences of assessing the students in the digital OSCE. Using content analysis, their responses were categorized into two over-arching themes. One theme concerned positive aspects of the digital OSCE, comprising four sub-themes, and a second theme concerned negative aspects of the digital OSCE, comprising three sub-themes.

### Positive Aspects of the Digital OSCE

#### Worked Out Well

Thirteen of the examiner responses expressed that the digital OSCE worked out rather well in general, that the SPs did a good job, and that the technology worked well.

#### Practical Advantages

Nine of the examiner responses concerned practical advantages with the digital OSCE such as less logistical requirements, more time efficient and more time at each station for the students. Two of the examiner responses also expressed the benefit of being able to deliver a digital OSCE during the pandemic when the onsite OSCE could not be used. Further, five of the comments concerned that the examiners felt they were more invisible in the digital OSCE as they could turn off the camera and microphone in Zoom while observing the student interacting with the SP. When they turned off the camera and the microphone, they felt that they could more easily shift between observing the student and writing comments without disturbing the student.

#### Possibilities to Include New Content

One comment highlighted that in a digital OSCE, skills related to internet therapy could more easily be incorporated, which would be in line with contemporary development of clinical psychology practice.

#### Less Stress and Worry for Students and Examiners

Six of the comments revealed that the examiners thought that the digital OSCE was less stressful for the students compared to the onsite OSCE. One suggested reason was that feelings of stress and anxiety among students would not spread in the student group, which could be the case in an onsite OSCE. Another suggested reason was, that the student couldn’t see the examiner in Zoom and that students might feel more relaxed taking the OSCE in their home-environment. Three comments concerned that for the examiners themselves, the digital OSCE was less stressful, and that it was easier to stay focused during the OSCE.

### Negative Aspects with the Digital OSCE

#### Worries About IT and Technology

Six of the comments focused on the stress the examiners felt before the OSCE that was related to the risk of IT-problems during the digital OSCE. Two of the comments also mentioned that there were some cases of IT-problems during the examination.

#### Fatigue

Five comments addressed feelings of an increased fatigue related to assessing students in Zoom, compared to in an onsite face-to-face situation. One examiner commented on that this fatigue might affect the SPs as well.

#### Reliability and Validity of the Digital OSCE

Twelve of the examiner comments concerned the difficulty of assessing relational aspects between the student and the SP in the digital OSCE, for example to assess empathy and to observe non-verbal behavior such as body language, mimic in the interaction between student and SP. One examiner also commented that due to these limitations, some of the situations also become less authentic, and that some of the stations, for example diagnostic assessment and PDT treatment, are more difficult to assess in a digital OSCE. The difficulties of communicating with non-verbal signals are also something that can make it more challenging for the students to make a proper judgement of the SP. Two of the examiner comments concerned that more than five stations are needed to make a holistic judgement of a students’ clinical skill and ability. Four of the comments also expressed concerns related to the inter-rater reliability as there were parallel circuits and two examiners were assessing the same stations. Finally, one examiner commented on the fact that more time for the teacher team would be needed for developing new OSCE stations and evaluate previous OSCEs.

## Discussion

Assessing complex clinical skills in psychology in a reliable and valid manner is a challenge. Recently, researchers in psychology have advocated a greater use of the OSCE for such assessments (Yap et al., 2021). During the covid-19 pandemic, there was an increased need for digital assessment alternatives, and a digital OSCE was implemented at the Programme for Master of Science in Clinical Psychology, at Umeå University, Sweden. The present study evaluated the digital psychology OSCE, with respect to students’ and examiners’ perceptions.

In order to assess students’ perceptions of the OSCE, a modified questionnaire developed by Yap et al. (2012) and later modified by Sheen et al. (2015) was used. The items and sub-scales in the student questionnaire were subjected to psychometric analyses. The results showed that most items exhibited acceptable levels of item-discrimination. In the subscales of feelings before and during the OSCE, three of the four items with low levels of item-discrimination were positively worded items (*felt excited, felt sure I would pass, highly focused*). With respect to internal consistency, all subscales also demonstrated acceptable or close to acceptable levels. Taken together, the result from the psychometric analyses indicate that the items in the student questionnaire are reliably assessing different aspects of student perceptions of the OSCE. Further modifications of the instrument could include altering the positively worded items or adding more to form a separate subscale of positive OSCE experiences.

### Students’ Feelings About and Perceptions of the OSCE

Almost 60 percent of the students in the present study reported that they felt stressed, and over 75 percent felt nervous during the digital OSCE. In line with previous studies, this indicates that OSCE in clinical psychology is to be considered a stressful examination form (Johnson et al., 2018; Sheen et al., 2015; Yap et al., 2012). To increase the knowledge of both positive and negative aspects of students’ feelings about the test situation, some items were added to the questionnaire in the present study. The results showed that before taking the OSCE about 70 percent of the students worried about the difficulty level and about 65 percent felt afraid to fail, which indicates that students believed that the OSCE would be quite a difficult examination. Similar results were also found by Dunsmuir et al. (2020) where students reported anticipatory anxiety before the OSPA. One explanation to these feelings of worry might be unfamiliarity with the OSCE format, an explanation that is supported by findings in previous studies where factors contributing to the anxiety included the build-up prior to the assessment day, and the experience of the OSCE being new and unknown to the students (Dunsmuir et al., 2020; Yap et al., 2012). Another explanation for the perceived stress is related to the finding that nearly 90 percent of the students consider the OSCE to be a more important examination than other examinations, which emphasizes the high-stakes character of the OSCE. In this perspective, the stress associated with the OSCE does not necessarily need to be a disadvantage but can be considered beneficial in terms of the authenticity of the examination, preparing student for clinical practice (Sheen et al., 2015).

Although the OSCE was highly associated with stress and anxiety, nearly 60 percent also felt excited before the OSCE, indicating a positive anticipation and implying high motivation. Moreover, the results show that most of the students felt concentrated, and about 60 percent of the students reported that they felt highly focused during the OSCE, which suggests that for these students the level of stress did not affect their performance to a significant extent. The feelings of excitement, concentration and high focus are particularly interesting, as these aspects of students’ experiences do not seem to have been explored in previous studies of OSCE in psychology.

Nevertheless, it is worth noting that a small group of students reported that they felt mentally blocked during the examination, and this could of course very well have detrimental effects on their test-performance, which in turn would have negative impact on the validity of their scores (Crooks et al., 1996). In order to avoid these validity threats, strategies of modifying OSCE need to be considered. These concerns have been addressed in previous studies, with mixed results (Johnson et al., 2018; Yap et al., 2012). Introducing practical sessions, as well as making stations longer, did not decrease the level of stress. However, improving information and instructions to students have in some cases been proven helpful (Johnson et al., 2018). One could argue that information clarity is an important issue in any examination, and therefore always worth reviewing.

Students were also asked about the OSCE as a learning experience and about the quality and validity of the OSCE. The results showed that they perceived the learning experience of both the preparatory course and the OSCE as very positive, and they believed that the feedback from the OSCE helped increase their self-awareness of their professional skills. These findings are in line with previous studies of OSCEs in psychology, where results have shown that students believe that the OSCE is a useful learning experience, and that feedback from examiners helped facilitate learning (Dunsmuir et al., 2017; Johnson et al., 2018; Sheen et al., 2015; Yap et al., 2012). Moreover, most students reported that the OSCE was a valid assessment for examining clinical skills, and that the stations were relevant and the SPs were realistic. The students also rated the fairness, relevance and quality of the OSCE as high in general, in line with earlier research on onsite psychology OSCEs (Sheen et al., 2015; Yap et al., 2012).

### Examiners’ Perceptions of the Quality and Validity of the OSCE

In line with previous studies of onsite OSCEs, the examiners in the present study perceived the digital OSCE as a fair and relevant examination and a quality-enhancing element in the psychologist education (Johnson et al., 2018; Sheen et al., 2015). Further, they rated the OSCE to be a valid examination form for assessing general skills and abilities. However, concerning more specific skills, they used the “unable to assess” alternative to a high extent indicating that it might be hard for examiners to evaluate the skills they are not personally familiar with. When the examiners were asked to comment on their experiences of the digital OSCE, they addressed some interesting points. Although most examiners thought that the digital OSCE worked out well, they identified some drawbacks as well. For example, they commented on that more than five stations would be needed to make a holistic judgement of the students’ clinical skills and abilities. This comment is relevant since it has previously been pointed out in the literature that OSCE has increased reliability and content validity with a greater number of stations (Kaslow et al., 2009). However, as has been highlighted in several studies, arranging large OSCEs is a costly and resource demanding undertaking (see e.g. Sheen et al., 2015). For this reason, the use of different modalities for stations, such as video simulation and written cases, can be considered as possible cost-saving measures instead of cutting down on the number of stations (Yap et al., 2021). The digital OSCE in the present study allowed for parallel rounds. In relation to this, the examiners raised the question of inter-rater reliability. Calibrating examiners through training and examining the inter-rater reliability is an important quality measure for OSCEs with parallel circuits (Yap et al., 2021). Moreover, the examiners commented on the difficulty of assessing relational aspects, and non-verbal communication in the digital OSCE, which is an important validity issue. Since displaying and expressing empathy and collaborating with the patients are some of the core competencies that the OSCE was meant to target, this must be considered carefully.

Another problem identified by the examiners concerned that it is quite taxing to assess students in a digital OSCE compared to onsite. A longer OSCE would have been even more demanding on the examiners. A perhaps less taxing alternative to having one high-stake digital (or onsite) OSCE with many stations could be to have multiple OSCEs with few stations, as suggested by Blythe et al. (2021) and Yap et al. (2021). This could also be beneficial from the perspective of covering more of the core competencies, less demanding from an administrative point of view, and could also lead to less vulnerability to unforeseen events. From a student perspective, participating in OSCEs repeatedly could also lower the stress, due to increased familiarity of the assessment.

The examiners also identified positive aspects of the digital OSCE, such as technology working well, that they as examiners became more invisible, and that this might reduce stress for students. They also believed that the level of stress for students was reduced in the digital OSCE since feelings of stress do not spread between students as they may do in an onsite OSCE. This hypothesis, however, would have to be further investigated.

### Limitations and Future Research

There are some methodological considerations of the present study that are worth noting. The use of digital OSCEs is new in psychology, and as far as we know this study is probably one of the first to evaluate student and examiners perception of this new digital assessment method. Although results provided a positive picture of digital OSCE in psychology in general, the samples of students and examiners participating in this study were rather small, based on only two cohorts of students and examiners from two OSCE occasions. In addition, some examiners participated on both OSCE occasions, but due to the anonymous data it was not possible to match responses for the same examiners from the different OSCE occasions. Due to these limitations, more and larger studies are needed to evaluate the generalizability of the findings. Moreover, as participation in the study was voluntary, self-selection might have biased the sample towards positive views of the OSCE. This study indicates that the digital OSCE works rather well from a student and examiner perspective, while some issues were noted. Although the questionnaires used in the present study are based on previously used OSCE questionnaires (Sheen et al., 2015; Yap et al., 2012), the reliability and validity of these have not been reported on. In the present study, initial psychometric analyses were conducted on the student questionnaire, and overall results indicated acceptable item and scale reliabilities. Because of the small sample size and partial missing values due to the option “unable to assess” a similar evaluation of the examiner questionnaire was not possible to conduct in the present study. Therefore, an important topic of future studies would also be to examine the psychometric quality of the examiner questionnaire. In order to further examine the functioning of the digital OSCE, psychometric studies on OSCE performance data, including examination of the inter-rater reliability, would also be required. The onsite Psychology OSCE at Umeå University has recently been subject to psychometric evaluation (Sundström & Hakelind, 2022). Future research should include similar studies on the digital OSCE.

Despite these limitations, the present study has important contributions. First, the implementation of the OSCE in Psychology is a rather novel phenomenon, in particular the use of digital OSCE. Previous studies about onsite psychology OSCEs (Sheen et al., 2015; Yap et al., 2012) have indicated that students and examiners judge the OSCE to be a fair, valid and high-quality examination-form for assessing clinical skills in psychology. The present study indicates that this may also be the case for a digital OSCE. However important questions were raised by examiners regarding the difficulty to accurately assess relational competences in the digital format. This is an important aspect to consider when planning for future OSCEs in Psychology.

## Conclusions and Practical Implications

The covid-19 pandemic challenged us, forced us to find new creative digital solutions to be able to maintain high quality assessments in the universities all over the world (Montenegro-Rueda et al., 2021). The use of digital examinations has increased rapidly in higher education during the pandemic (Senel & Senel, 2021). This development is likely to influence future examinations, why it is likely that digital OSCEs will continue being used, perhaps also in a growing number of psychology educations. This is a likely development especially in view of the practical advantages of less logistical requirements compared to an onsite OSCE. The present study examined students’ and examiners’ perceptions of a digital OSCE in psychology. Students generally perceived the digital OSCE as a positive and valuable experience. Although students worried about the OSCE and perceived it as a stressful examination, most of them still felt concentrated and focused during the OSCE, which indicates that the level of stress did not affect their performance to a large extent. Moreover, students and examiners generally found the digital OSCE to be a relevant, authentic and fair examination and a quality enhancing element in psychology education. Based on the results from the present study some future directions for digital OSCEs can be suggested. Overall, it seems that digitalizing the OSCE can work well, but the results also indicate that it is difficult to assess certain aspects digitally, such as some of the relational skills, which are central to the psychologist profession. It was also pointed out that a limited number of stations is problematic, which is supported in the literature as reliability and content validity increases with a higher number of stations (Kaslow et al., 2009). These issues need to be considered and addressed when planning future digital OSCEs.

## Supplemental Material

sj-docx-1-plj-10.1177_14757257221114038 - Supplemental material for Examining Skills and Abilities During the Pandemic – Psychology Students’ and Examiners’ Perceptions of a Digital OSCE Supplemental material, sj-docx-1-plj-10.1177_14757257221114038 for Examining Skills and Abilities During the Pandemic – Psychology Students’ and Examiners’ Perceptions of a Digital OSCE by Camilla Hakelind and Anna E. Sundström in Psychology Learning & Teaching
