# Supplementary material for: Examining Skills and Abilities During the Pandemic – Psychology Students’ and Examiners’ Perceptions of a Digital OSCE
Source: Psychol Learn Teach. 2022 Jul 22;21(3):278–95. doi: 10.1177/14757257221114038 (PMC9309587; doi:10.1177/14757257221114038)
Supplement: sj-docx-1-plj-10.1177_14757257221114038 - Supplemental material for Examining Skills and Abilities During the Pandemic – Psychology Students’ and Examiners’ Perceptions of a Digital OSCE [file sj-docx-1-plj-10.1177_14757257221114038.docx]

**Appendix A: Example material for one station in the digital OSCE. The example show material that can be used for a CBT station examining students’ ability to perform a functional analysis.**

1. Example of instructions to student:

You are working at a health care clinic and have met Alex, who is 48 years old, on two occasions before. Alex has sought help for her lack of energy and vigor, which has contributed to an experience of depression. Above all Alex is tormented by passivity and recurring depressive thoughts. Alex is living alone; her adult son and little grandchild are living abroad and they have not been able to meet in a very long time, due to the Covid-19 restrictions. Her work has earlier been a source of stimulation and social interaction, but Alex has the last six months had to work from home and has thus not met her colleagues other than sporadically. Summer became rainy and Christmas was spent alone. Now she finds it very difficult to motivate herself to get up in the mornings and manage her work. She often doesn’t answer when friends call. You have already worked on mapping her life situation and agreed to examine more specifically which circumstances that contribute to maintaining her passivity and depression. When you now meet Alex again, your plan is to make a functional analysis of one or more of Alex's behaviors.

To do:

• Give a rationale for today's session (explain the purpose of what you are going to do)

• Make a functional analysis of the client's problem behavior/problems

• Include relevant respondent reactions

• Include relevant operant behaviors

• Inform the client about how this can be used in treatment

1. Example of material for assessment

Table showing typical example of checklist for assessment. Maximum score is 10 points.

| *Assessment criteria* | *Points* | *Remarks* |
| --- | --- | --- |
| The student shows alliance building behavior |  |  |
| The student gives a rationale for functional analysis |  |  |
| The student keeps the structure of the session |  |  |
| The student explores thoughts, feelings and bodily reactions |  |  |
| The student explores relevant respondent behaviors |  |  |
| The student explores relevant operant behaviors |  |  |
| The student explains how the functional analysis can be used in treatment |  |  |

Table showing the global rating scale

| *Global rating scale* |  |
| --- | --- |
| Excellent |  |
| Pass |  |
| Borderline fail |  |
| Clear fail |  |

1. Example of typical instructions to actor playing the standardized patient:

Your name is Alex and you are 48 years old. You have met the psychologist at your health care center on two occasions before. Today you have agreed to examine more specifically in which situations you have the most difficulties. You have sought help for your lack of strength and energy, which has contributed to an experience of depression. Above all, you are struggling with passivity, and recurring depressive thoughts. You are living alone, and your adult son and small grandchild live in the US. You have not been able to meet with them for a year due to the covid-19 pandemic. You miss the son and the little grandson who is two and a half years old. Previously, you have been able to visit at least twice a year and the son and family have come home to visit every summer.

Working as an administrator at a government agency has earlier been something you’ve found stimulating, and you used to enjoy the social interaction with your colleagues, but in the last six months you have had to work from home and have therefore not met your colleagues other than sporadically. The summer became rainy, and you did not come to do much during the holidays. Now you have a very hard time motivating yourself to get up in the mornings and get your work started. You often wake up early but stay in bed. You do not answer the phone as often when friends call anymore.

You are a little surprised by the depression you notice, since you have not felt this way before in your life. You have had a few minor life crises, but they have always passed. You feel a little ashamed of your situation and do not want to show friends, neighbors and co-workers how you feel. You sleep somewhat irregularly, have difficulty eating regularly and do not engage in any regular physical activity. You are somatically healthy and have had a medical checkup a few months before you decided to go to the psychologist. In the earlier sessions, you and the psychologist worked with mapping your life situation, and at the last visit the both of you agreed to investigate more specifically which contexts that contribute to maintaining passivity and depression.

Specific instructions on how to answer and react:

- Be careful not to give too much information at a time - the psychologist must work on his questions to get the information needed.
- But you should also not be "too difficult". When asked, do answer.
- The student will probably focus on a situation where your problems show up. The student should ask you about one or two situations and will probably ask you to describe more about them.
- It is therefore essential that you are prepared to answer questions about a couple of specific situations, related to the case, that you feel are difficult for you and where you react with avoidance and passivity, or situations that make you start thinking depressive thoughts.
- Be prepared to answer questions about how you react, how you think, and how you feel in these situations, both regarding emotions and bodily reactions, depending on what the student asks you about.
- Before starting the OSCE, you will discuss suitable situations, feelings, thoughts and reactions more in detail with the expert/examiner.
